# The effects of active workstations on reducing work-specific sedentary time in office workers: a network meta-analysis of 23 randomized controlled trials

**DOI:** 10.1186/s12966-023-01467-5

**Published:** 2023-07-27

**Authors:** Liying Zhou, Xinxin Deng, Meng Xu, Yanan Wu, Xue Shang, Fenfen E, Yongsheng Wang, Shanshan Liang, Kehu Yang, Xiuxia Li

**Affiliations:** 1 Health Technology Assessment Center/Evidence-Based Social Science Research Center, School of Public Health, Lanzhou University, 199 Donggang West Road, Lanzhou, 730000 China; 2 Evidence Based Medicine Center, School of Basic Medical Sciences, Lanzhou University, 199 Donggang West Road, Lanzhou, 730000 China; 3 Key Laboratory of Evidence Based Medicine and Knowledge Translation of Gansu Province, Lanzhou, 730000 China

**Keywords:** Active workstation, Workplace, Sedentary behavior, Network meta-analysis

## Abstract

**Background:**

Active workstations have been proposed as a feasible approach for reducing occupational sedentary time. This study used a network meta-analysis (NMA) to assess and compare the overall efficacy of active workstation interventions according to type and concomitant strategy for reducing work-specific sitting time in office workers.

**Methods:**

PubMed, Web of Science, EMBASE, and Cochrane Central Register of Controlled Trials (CENTRAL) databases were searched from database inception until May 2022 to obtain randomized controlled trials (RCTs) assessing the efficacy of active workstations with or without concomitant strategies for reducing occupational sedentary time in office workers. The risk of bias of the RCTs included in this study was assessed according to the Cochrane Handbook. An NMA with STATA 15.1 was used to construct a network diagram, league figures, and the final surface under the cumulative ranking curve (SUCRA) values. The certainty of evidence was assessed using the grading of recommendations, assessment, development, and evaluation (GRADE) approach.

**Results:**

A total of 23 eligible studies including eight different types of interventions with 1428 office workers were included. NMA results showed that compared to a typical desk, multicomponent intervention (standardized mean difference (SMD) =  − 1.50; 95% confidence interval (CI) − 2.17, − 0.82; SUCRA = 72.4%), sit-stand workstation + promotion (Reminders of rest breaks, posture variation, or incidental office activity) (SMD =  − 1.49; 95%CI − 2.42, − 0.55; SUCRA = 71.0%), treadmill workstation + promotion (SMD =  − 1.29; 95%CI − 2.51, − 0.07; SUCRA = 61.6%), and sit-stand workstation (SMD =  − 1.10, 95%CI − 1.64, − 0.56; SUCRA = 50.2%) were effective in reducing occupational sedentary time for office workers.

**Conclusions:**

Multicomponent intervention, sit-stand workstation + promotion, treadmill workstation + promotion, and sit-stand workstation appear to be effective in reducing work-specific sedentary time for office workers. Furthermore, multicomponent interventions and active workstations + promotion better reduced work-specific sedentary time than active workstation alone. However, the overall certainty of the evidence was low.

**Trial registration:**

Our study protocol was registered with the International Prospective Register of Systematic Reviews (PROSPERO); registration number: CRD42022344432.

**Supplementary Information:**

The online version contains supplementary material available at 10.1186/s12966-023-01467-5.

## Background

Due to rapid advancements in science and technology, and the continuous mechanization, automation, and informatization of society, many labor jobs have transferred into the sedentary service industry and office-based professions, coinciding with decreased energy expenditure and fewer physical activity opportunities [1]. According to research findings, a notable proportion of the sedentary behavior of employed adults, ranging from approximately 40% to 70%, transpired during the course of their occupational duties [2, 3]. More sitting time was reported at work than for other sitting activity, such as watching television or using a computer at home on weekdays. Studies also revealed that full-time office workers’ working time sitting accounted for approximately 60% to 90% of the total daily sitting time on a work day [4, 5]. In addition, there is evidence that working adults spending long periods sitting at work do not necessarily compensate for their sitting at work by being more active outside of work [6]. It is crucial to note that contemporary research indicates that excessive sedentary behavior is detrimentally linked to many health risks, such as cardiovascular disease, unhealthy aging, musculoskeletal disorders, poor bone health, poor metabolic health, and all-cause mortality, especially when the sedentary time accumulates in prolonged uninterrupted bouts [7, 8]. The workplace has been highlighted by the World Health Organization as a vital setting for health promotion action to reduce sedentary behavior [9]. Therefore, targeted efforts to address sedentary behavior and excessive sitting time in the workplace are undoubtedly necessary for better health outcomes.

Recently, there has been interest in targeted interventions using active workstations in the office setting to address activity during working hours, such as sit-stand workstations, treadmill workstations, and cycling workstations [10]. Users are able to infuse movement into their workday through the assistance of these active workstations. For example, sit-stand workstations allow users to alternate between sitting and standing by lowering or raising the work surface. Treadmill workstations comprise a height-adjustable standing desk, as well as an under-desk treadmill, allowing users to walk slowly while simultaneously carrying out tasks at the computer. By using a treadmill workstation, individuals can break away from the sedentary lifestyle typically associated with office work and incorporate light exercise into their workday. Importantly, evidence has shown that compared with typical desks, active workstations can be effective to reduce occupational sitting time, maintain workforce performance, raise energy expenditure, regulate ambulatory blood pressure, increase attention and memory, and improve chronic low back pain [11, 12].

Based on the findings of two umbrella reviews, the utilization of electronic and mobile health tools, such as mobile applications, is associated with a reduction in sedentary behavior [13, 14]. In addition, the current umbrella reviews indicate that interventions targeting the physical environment, specifically the implementation of active workstations, represent the most efficacious category of interventions for mitigating sedentary behavior in workplaces [15, 16]. Considering the increasing public health attention regarding workplace sitting and non-manual employees’ interest for active workstations, identifying the most appropriate and effective active workstation interventions based on type and concomitant strategy is important. However, existing literature reviews have been limited in that context due to their focus on only a single active workstation intervention type, rather than comparing the effectiveness of various interventions in the workplace. In addition, these results have all been based on qualitative descriptions or direct comparisons in a few trials. Finally, there is no detailed classification of active workstations, which are varied across studies.

The network meta-analysis (NMA) is a type of meta-analysis that allows for the simultaneous comparison of multiple interventions using both direct and indirect evidence [17]. Its estimation of the relative effectiveness among all interventions and rank ordering of the interventions even if head-to-head comparisons are lacking. In comparison to other types of meta-analyses, NMAs have the advantages of synthesizing evidence from both direct and indirect comparisons, allowing for a comprehensive assessment of the available data. In our study, the NMA was used to integrate data from multiple trials and provide valuable insight into the effects of different types of active workstation interventions and concomitant strategies on reducing work-specific sedentary time in office workers. With the emergence of new trials and comparisons, the results of these studies should be updated and expanded. Citing newly published trials, this study aims to perform an NMA to identify the work-specific sedentary time reduction effects of different types of active workstation interventions and concomitant strategies for office workers.

## Methods

### Registration

The protocol was registered in the International Prospective Register of Systematic Review (PROSPERO) database on July 5, 2022 (registration number: CRD42022344432).

### Search strategy

A systematic search was performed in the PubMed, Web of Science, EMBASE, and Cochrane Central Register of Controlled Trials (CENTRAL) from database inception to May 17, 2022. The search strategies were developed by a senior reviewer (Xiuxia Li), and the detailed search strategy is presented in Additional file 1. The main search strategies were as follows: (occupation* or workplace* or employe* or office* or work-site or worker* or staff* or white-collar*) AND (sedentary or sitting or inactivity or “physical activity” or “physically active”) AND (random* or blind* or singleblind* or doubleblind* or tripleblind* or RCT* or control*). In addition, the WHO International Clinical Trials Registry Platform (ICTRP) search portal, ClinicalTrials.gov, and reference lists (backward and forward) of the studies identified using the above search strategy were searched manually for additional articles on May 17, 2022. We searched for the full texts identified by conference materials through Google Scholar. Full texts of conference papers that meet our inclusion criteria were included in the NMA. We also searched relevant grey literature including clinical guidelines, reports, and working papers through Google and grey literature database (http://www.opengrey.eu/).

### Inclusion and exclusion criteria

Studies published in English meeting the following criteria were included:

#### Participants

All studies involving office workers aged ≥ 18 years whose occupations involved spending the majority of their working time at a desk were eligible; examples include administrative workers, customer service operators, help-desk professionals, call-center representatives, and receptionists.

#### Interventions

We focused on the active workstations and concomitant strategies aimed at changing occupational sedentary behavior; examples include sit-stand desks, vertical workstations on treadmills, desk cycle/cycling desks, and under desk steppers.

#### Comparisons

No restrictions were placed on the comparison groups.

#### Outcomes

The outcomes were limited to work-specific sitting time reductions measured with objective parameters (e.g., accelerometry) or self-reporting (e.g., questionnaires and activity diaries) at primary time point.

#### Study design

Only studies with a concurrent control group for the interventions were included in this review; examples include randomized controlled trials (RCTs), cluster-RCTs, and quasi-experimental studies.

Studies were excluded if they (1) were two-arm trials investigating the effectiveness of different levels or durations of the same intervention without any additional interventions element, such as alternative interventions or (2) were duplicate publications, reviews, or protocols or had incomplete data.

### Literature selection and data extraction

**Fig. 1:**
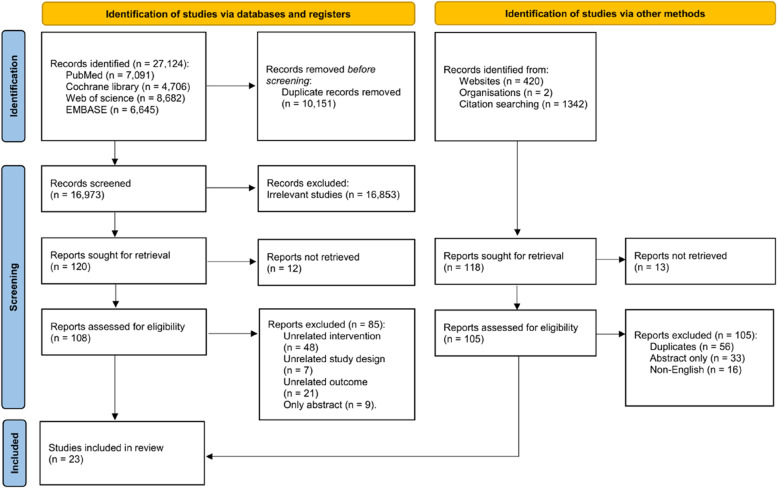
Flowchart of literature selection

Endnote X9.1 literature management software was used to manage the literature search records. To ensure high inter-rater reliability among the reviewers, a pilot-literature selection was performed. According to the inclusion and exclusion criteria, two independent reviewers (Liying Zhou and Xinxin Deng) screened the titles and abstracts of all retrieved studies for relevance after omitting duplicates; then, the reviewers scrutinized full-text articles whose abstracts were identified as relevant or potentially relevant. Each study was evaluated strictly against the pre-set criteria, and any disagreement regarding study inclusion was resolved by discussion with a third reviewer. We recorded the selection process in sufficient detail to complete a Preferred Reporting Items for Systematic Reviews and Meta-Analyses (PRISMA) flow diagram (Fig. 1) [18].

We extracted the following data from the included studies by using a pre-specified data form: general information (publication date, name of first author, study country/region), study population (age, sex, education, employment status), active workstation intervention (type, intervention frequency and duration, delivery mode, and theoretical framework), comparison intervention (wait list, no intervention, or other), occupational sitting time, and follow-up time. The data are presented as the mean ± standard deviation (SD); if the end-of-study values were not available, they were imputed according to the Cochrane Handbook.

### Risk of bias assessment

The Cochrane risk of bias tool was used to evaluate the quality of the RCTs; this method was based on randomization and allocation concealment (selection bias), blinding of the personnel and participants (performance bias), blinding of the outcome assessment (detection bias), incomplete outcome data (attrition bias), selection of the reported results (reporting bias) and sources (other bias) and indicates low, high, or unclear risk of bias [19]. Studies were rated as “low” risk of bias if all items were low risk. When one item was high, the study was rated as “high” risk of bias. For all other conditions, the studies were rated as “unclear” risk of bias. In all the included studies, blinding of personal and participants to the intervention and allocation concealment were not feasible due to the inherent nature and objective of the intervention, which involved changes in the environment such as the installment of sit-stand workstation. As a result, the performance bias item and allocation concealment item were excluded from the bias assessment. However, for the allocation concealment item, trials were evaluated based on the presence of contamination between participants in the intervention and control groups, i.e., individuals from the same office ended up in different groups, which can confound the results. Control group participants may be influenced by intervention group participants in the same office, regardless of group allocation, leading to potential bias [20–22]. Studies were considered to have a low risk of bias if measures were taken to minimize contamination, such as using cluster trials or assigning intervention and control participants to separate floor in the same building. Studies were rated as high risk of bias if intervention and control group participants were present in the same office setting. Studies were classified as unclear risk of bias if there was insufficient information to determine the presence of either of the above conditions. The risk of bias assessment was completed independently by the two reviewers. If discrepancies arose, the reviewers discussed the issue until a consensus was reached [23]. For studies with multiple publications, we reviewed all relevant papers, including the protocol paper, to ensure the quality of the trial was judged on all available information.

### Data analysis

We used STATA 15.1 software (the network package 18 and the network graphs package) to complete the NMA [17, 24–26]. First, the two reviewers categorized the interventions and extracted the sample sizes and work-specific sitting time reductions, to be used in the STATA network suite of commands. The reviewers resolved disagreements through discussion or through arbitration by a senior reviewer (Xiuxia Li). After data extraction, the data was set up using an augmented format where all treatments were compared with a reference treatment. The augmentation process using arm-based values calculated the risk of estimates of the comparisons with the reference treatment and their variances and covariances. We then generated a network map to determine if an NMA was feasible. A network diagram with nodes and lines was constructed to summarize the evidence. The sizes of the nodes show the number of populations of the studies, and the thicknesses of the lines between the nodes indicate the number of studies included [27]. After that, we performed an NMA within a frequentist framework using a multivariate random effects meta-analysis estimated by the restricted maximum likelihood. Direct comparisons were made when two interventions were compared head-to-head within a study, while indirect comparisons were made when treatments were not compared head-to-head but were compared through a common comparator. The NMA results were summarized based on all possible comparisons, including direct and indirect comparisons. Reduced occupational sitting time was a continuous variable, and the standard mean difference (SMD) and 95% confidence interval (CI) were used to estimate the effect size of the different comparisons; significant differences are indicated by a *P*-value of < 0.05 [28].

We performed an overall inconsistency test and used the P-value to determine the consistency level [29–31]. A P-value > 0.05 signifies a good consistency. If a closed loop connecting different interventions existed, a node-splitting test was used to assess the local inconsistency between direct and indirect comparisons. Differences between direct and indirect coefficients in terms of P-values were used to estimate the inconsistency. If P < 0.05, local inconsistency was considered to exist. Important inconsistencies can threaten the validity of the results; if present, the possible sources of disagreement were explored and identified.

Finally, to rank the probability of which intervention could reduce the occupational sedentary time best, we calculated the value of the surface under the cumulative ranking curve (SUCRA). SUCRA indicates the area under the curve of the cumulative ranking of probabilities for each intervention and is expressed as a percentage between 0% (i.e., the treatment always ranks last) and 100% (i.e., the treatment always ranks first). A higher SUCRA value indicates that the higher probability of an intervention being the best. SUCRA is an index that can be used as a reference to evaluate the relative position of each treatment and account for inconsistencies between studies [32, 33].

### Certainty of evidence

We rated the certainty of evidence for each network estimate using the GRADE framework, which classifies evidence as high, moderate, low, or very low certainty. The starting point for certainty in direct estimates for RCTs is high but can be downgraded based on limitations for risk of bias, imprecision, inconsistency (heterogeneity), indirectness, and publication bias. Judgements for each factor can be ‘not serious’ (not degraded), ‘serious’ (degraded by one level), or ‘very serious’ (degraded by two levels) [34, 35].

We rated the certainty of evidence for each direct comparison according to standard GRADE guidance for pairwise meta-analyses. Indirect effect estimates were calculated from available “loops” of evidence, which included first order loops (based on a single common comparator treatment; that is, the difference between treatment A and B is based on comparisons of A and C as well as B and C) or higher order loops (more than one intervening treatment connecting the two interventions). We assessed the evidence for indirect and network estimates focusing on the dominant first order loop and rated the certainty of indirect evidence as the lowest certainty of the direct comparisons informing that dominant loop. In the absence of a first order loop, we used a higher order loop to rate the certainty of evidence and used the lowest of the ratings of certainty for direct estimates contributing to the loop. We considered further downgrading each indirect comparison for intransitivity if the distribution of effect modifiers differed in the contributing direct comparisons.

For the network estimate, we started with the certainty of evidence from the direct or indirect evidence that dominated the comparison and, subsequently, considered downgrading our certainty in the network estimate for incoherence between the indirect and direct estimates for imprecision (wide credible intervals) around the treatment effect estimates. When serious incoherence was present, we used that with the higher certainty of direct and indirect evidence as the best estimate.

## Results

### Literature screening process and results

A total of 27,124 potentially relevant studies were returned by the electronic searches. After screening the titles and abstracts, 108 were potentially eligible for full-text review. Ultimately, 23 studies reporting RCTs were eligible (Fig. 1) [20, 36–57]. We found no eligible articles through our supplemental search.

### Characteristics of the included studies

Table 1 shows an overview of the 23 RCTs included in this network meta-analysis reporting comparisons of one or more of the following components: sit-stand workstations, typical desks, exercise, promotion, treadmill workstations, seated ellipticals, and multicomponent interventions. Multicomponent interventions included individual (e.g. coaching, promotion), environmental (e.g. sit-stand workstations, work environment changes), and organizational components (e.g., ambassador management role, education workshop). In the included studies, the environmental modification strategy of multicomponent interventions necessarily included the installation of the active workstations. For the promotion intervention participants were reminded of rest breaks, posture variation, or incidental office activity via text messages, emails, apps, etc. The sit-stand workstation + promotion intervention comprised joint installment of a sit-stand workstation and participants reminders of rest breaks, posture variation (including increasing the use of the sit-stand intervention), or incidental office activity via messages, emails, phone apps, etc. The studies were published between 2012 and 2021 and included a total of 1428 participants (range: 15 to 231). Regarding the study locations, 11 were in Australia, three each were in the USA and England, two were in Canada, and one each was in Sweden, Finland, Japan, and Switzerland. The participants were mainly middle-aged people between 35 and 45. The primary endpoints of the included studies, the results of which were analyzed in this network analysis, ranged from one week to six months. A total of 21 studies used device-based measures, and two used self-reported measures as outcomes. The lowest dropout rate in the study was 0%, and the highest was 27.91%.

**Table 1:** Basic characteristics of included studies

First author (year)	Intervention	Sample (n)	Study design	Age	Type of work	Follow-up point ^a^	Dropouts ^b^	Measurement tool
Alkhajah (2012) [36], Australia	Sit-Stand Workstation	18	Quasi-RCT	33.5 ± 8.7	Student, general staff and academic staff of public health research centers within academic institutions	1-week, 3-month	3.13%	ActivPAL3 activity monitor
	Typical Desk	13		39.9 ± 7.2				
Bergman (2018) [20], Sweden	Treadmill Workstation + Promotion	39	RCT	52.4 ± 6.8	Staff of private companies, the government, municipalities, and counties	2-month, 6-month, 10-month, 13-month	1.25%	ActivPAL3 or activPAL3 micro activity monitor
	Sit-Stand Workstation	40		50.3 ± 6.7				
Carr (2016) [37], USA	Seated Elliptical + Promotion	27	RCT	45.2 ± 10.9	Staff of a private company	16-week	10.00%	GENEActiv Original accelerometer
	Promotion	27		45.0 ± 10.7				
Chau (2014) [38], Australia	Sit-Stand Workstation	18	Crossover-RCT	38 ± 11	Staff of a non-government health agency	4-week	0.00%	ActivPAL3 activity monitor
	Typical Desk	18						
Chau (2016) [39], Australia	Sit-Stand Workstation + Promotion	7	Quasi-RCT	31.0 ± 10.0	Customer care (call center) staff	1-week, 4-week, 19-week	21.05%	ActivPAL inclinometer and ActiGraph accelerometer
	Typical Desk	8		35.1 ± 11.5				
Donath (2015) [40], Switzerland	Sit-Stand Workstation + Promotion	15	RCT	45 ± 12	Staff of a health insurance company	12-week	18.42%	ActiGraph wGT3X-BT
	Sit-Stand Workstation	16		40 ± 10				
Dutta (2014) [41], USA	Sit-Stand Workstation + Promotion	14	Crossover-RCT	40.4	Staff of a private company	4-week	0.00%	Modular Signal Recorder 145 accelerometer
	Typical Desk	14						
Edwardson (2018) [42], England	Multicomponent Intervention	77	Cluster RCT (37 clusters)	41.7 ± 11.0	Staff of three university hospitals	3-month, 6-month, 12-month	23.29%	ActivPAL3 micro activity monitor
	Typical Desk	69		40.8 ± 11.3				
Mantzari (2019) 50, England	Sit-Stand Workstation	9	RCT	43.4 ± 11.2	Staff working full-time in professional job roles or positions involving clerical and administrative support	3-month	10.00%	ActivPAL3 activity monitor
	Typical Desk	9						
Engelen (2019) [43], Australia	Multicomponent Intervention	30	Quasi-RCT	44.48	Staff of a public transport organization	6-week, 13-week	10.87%	ActiGraph activity monitor
	Typical Desk	16						
Gao (2016) [44], Finland	Sit-Stand Workstation	24	Quasi-RCT	47.8 ± 10.8	Staff of a university	6-month	0.00%	Questionnaire
	Typical Desk	21		39.0 ± 8.5				
Graves (2015) [57], England	Sit-Stand Workstation	23	RCT	38.8 ± 9.8	Staff of a university	4-week, 8-week	0.00%	Ecological momentary assessment diaries
	Typical Desk	21		38.4 ± 9.3				
Healy (2013) [45], Australia	Multicomponent Intervention	22	RCT	42.4 ± 10.6	Staff of a government agency	4-week	16.28%	ActivPAL3 activity monitor
	Typical Desk	21		42.9 ± 10.3				
Healy (2016) [46], Australia	Multicomponent Intervention	136	Cluster RCT (24 clusters)	44.6 ± 9.1	Staff of government agencies	3-month, 12-month	21.74%	ActivPAL3 activity monitor
	Typical Desk	95		47.0 ± 9.7				
Johnston (2019) [47], Australia	Sit-Stand Workstation + Exercise	13	RCT	39 ± 11	Staff of universities	2-week, 4-week	10.34%	ActivPAL3 activity monitor
	Sit-Stand Workstation	13		40 ± 11				
Ma (2021) [48], Japan	Sit-Stand Workstation + Promotion	37	RCT	46.22	Staff of the technical department and general affairs department in a private company	4-month	0.00%	Active Style Pro HJA-750C accelerometer
	Typical Desk	38		44.6				
MacEwen (2017) [49], Canada	Sit-Stand Workstation	15	RCT	43.2 ± 9.7	Not report	12-week	0.00%	ActivPAL inclinometer and ActiGraph accelerometer
	Typical Desk	10		48.9 ± 11.4				
Neuhaus (2014) [51, 58], Australia	Multicomponent Intervention	16	RCT	37.3 ± 10.7	Staff of a university	3-month	9.09%	ActivPAL3 activity monitor
	Sit-Stand Workstation	14		43.0 ± 10.2				
	Typical Desk	14		48.0 ± 11.6				
Parry (2013) [52], Australia	Sit-Stand Workstation + Promotion	19	RCT	43.5 ± 6.4	Staff of three government agencies	12-week	27.91%	ActiGraph activity monitor
	Promotion	43						
Schuna (2014) [53], USA	Treadmill Workstation + Promotion	15	RCT	40.0 ± 9.5	Staff of a private health insurance company	3-month	0.00%	ActiGraph activity monitor
	Typical Desk	16		40.3 ± 10.9				
Stephens (2019) [54], Australia	Multicomponent Intervention	114	Cluster RCT (14 clusters)	44.9 ± 8.9	Staff of government agencies	3-month	0.00%	ActivPAL3 activity monitor
	Typical Desk	82		45.9 ± 9.8				
Tobin (2016) [56], Australia	Sit-Stand Workstation	18	RCT	34.8 ± 10.5	Staff of a non-government organization and a university	5-week	0.00%	ActivPAL3 activity monitor
	Typical Desk	19		34.3 ± 8.9				
Weatherson (2020) [55], Canada	Sit-Stand Workstation	17	RCT	40.96 ± 10.82	Staff of a university	3-month, 6-month	0.00%	ActivPAL3 activity monitor
	Typical Desk	20		37.24 ± 12.51				

*RCT* Randomized controlled trial; Table 1 provides a detailed description of the results at the primary endpoint, which were analyzed in the meta-analysis when there were multiple assessment points (a). Additionally, the dropout rate at the primary endpoint is also presented (b).; The descriptive statistics for age (in years) are presented as the mean ± standard deviation (SD) values

### Results of risk of bias

**Fig. 2:**
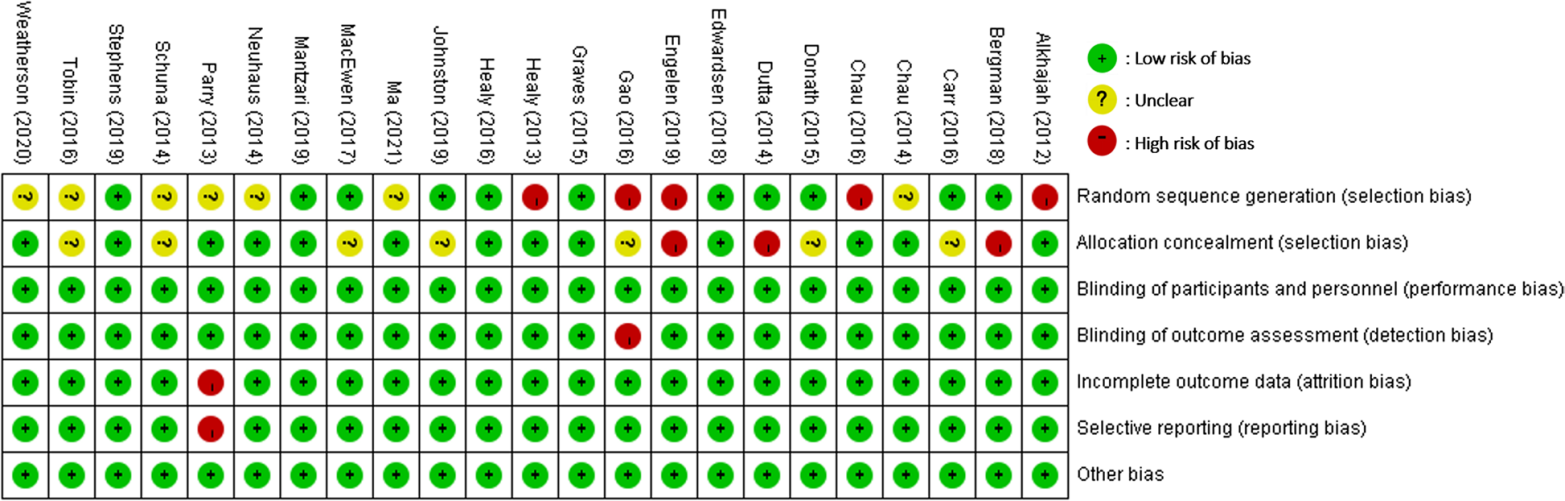
Risk of bias summary: Review of authors’ judgements about each risk of bias item for each included study with low, unclear, and high risk of bias for each feature from the Cochrane Risk of Bias Tool

As shown in Fig. 2, the risk of bias was assessed high in eight studies, unclear in ten, and low in five. Regarding the random sequence generation assessment, five studies did not adhere to random sequence generation, and thus we judged them to have a high risk of bias. Additionally, seven trials were assessed as unclear risk of bias because it gave no information about randomization was done. For allocation concealment, five trials were assessed as high risk of bias due to contamination between the intervention and control group participants, i.e., participants from the same office were placed in different group. Furthermore, seven trials were assessed as unclear risk of bias owing to insufficient information regarding contamination. Regarding outcome assessment, one trial was rated as high risk of bias because of its utilization of self-reported outcome measures. Regarding incomplete outcome data, one study was assessed as high risk of bias due to attrition rates exceeding 25%. Finally, concerning the selection of reported results, one trial was assessed as high risk of bias due to a lack of prospective registration.

### Network diagram

**Fig. 3:**
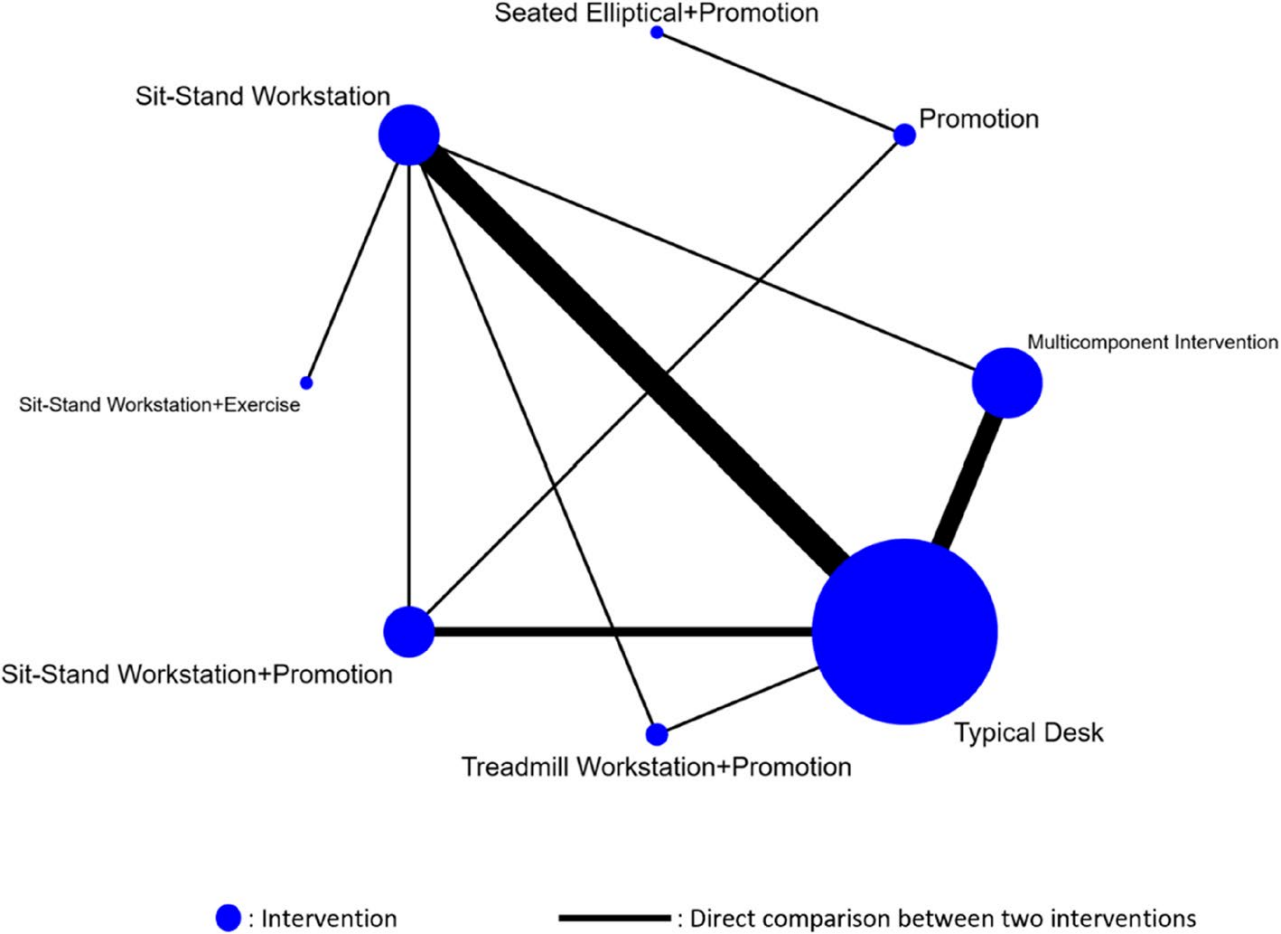
Network diagram of different interventions for work-specific sitting time reduction in office workers. The sizes of the nodes and the thicknesses of the edges are weighted according to the number of studies evaluating each treatment and direct comparisons, respectively

A network diagram was constructed based on the eight interventions: sit-stand workstation, typical desk, promotion, multicomponent intervention, sit-stand workstation + exercise, sit-stand workstation + promotion, seated elliptical + promotion, and treadmill workstation + promotion. A total of 10 direct comparisons and 18 indirect comparisons are included in this diagram (Fig. 3). The most comparisons were made for sit-stand workstations versus typical desks (reported by nine RCTs). Six and three RCTs compared the effect of a multicomponent intervention versus a typical desk and sit-stand workstation + promotion versus a typical desk, respectively. The remaining comparisons were each made in only one trial. Of the 23 studies, only four compared the effects of active workstations and concomitant strategies with active workstations alone. Furthermore, within the network of included studies, there were five closed loops connecting different interventions. The typical desk groups accounted for the largest sample size (*n* = 504), followed by multicomponent Intervention (*n* = 395), sit-stand workstations (*n* = 225), sit-stand workstation + promotion (*n* = 92), treadmill workstation + promotion (*n* = 70), promotion (*n* = 54), seated elliptical + promotion (*n* = 27), sit-stand workstation + exercise (*n* = 13).

### Inconsistency analysis

The global and local inconsistency test was to determine the consistency level. All fitted models converged well, and there was no evidence to indicate statistical inconsistency in our NMA (Additional file 2).

### NMA results

**Fig. 4:**
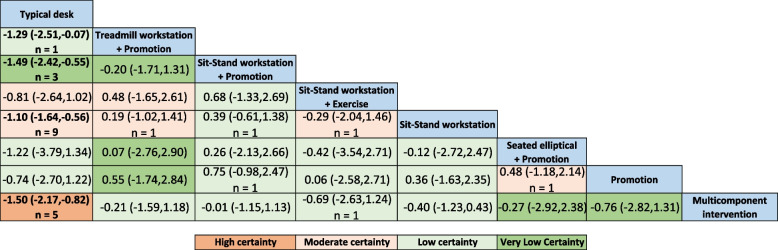
Network meta-analysis results with corresponding GRADE (grading of recommendations, assessment, development, and evaluation) certainty of evidence for work-specific sitting time reduction. Values correspond to the standardized mean difference (SMD) in work-specific time reduction between columns and rows: for negative values, the intervention indicated in the row may be better for reducing work-specific sitting time (e.g., the sit-stand workstation group had a work-specific sitting time reduction compared with the typical desk group; SMD =  − 1.10). Values in bold indicate a statistically significant treatment effect. N indicates the number of studies used for the comparison. Results without N in the boxes are derived through the process of indirect comparison in network meta-analysis

The results of the NMA are shown in Fig. 4. The final network effect showed that compared to typical desks, the interventions that effectively reduced work-specific sedentary time were sit-to-stand workstation (SMD =  − 1.10; 95%CI − 1.64, − 0.56), sit-to-stand workstation + promotion (Reminders of rest breaks, posture variation, or incidental office activity) (SMD =  − 1.49; 95%CI − 2.42, − 0.55), treadmill workstation + promotion (SMD =  − 1.29; 95%CI − 2.51, − 0.07), and multicomponent interventions (SMD =  − 1.50; 95%CI − 2.17, − 0.82).

### Probability ranking

**Fig. 5:**
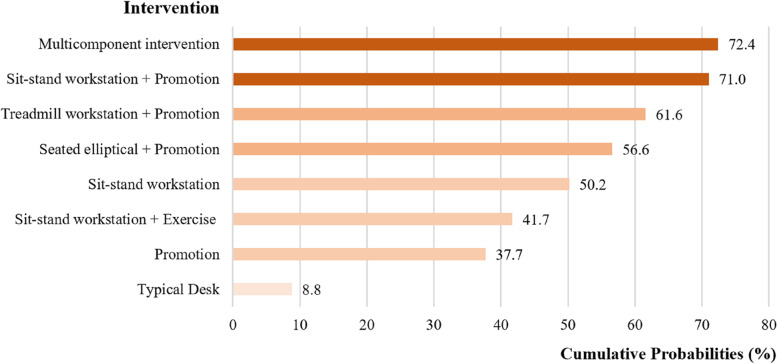
Probability ranking of all interventions according to the surface under the cumulative ranking curve results for reducing sedentary behavior in the workplace. A higher value indicates a higher probability of an intervention being the most effective

As presented in Fig. 5, the SUCRA probability ranking revealed different intervention effects. The effect of these seven interventions, ranking from highest to lowest most likely to be optimal intervention, were as follows: multicomponent interventions (SUCRA = 72.4%), sit-stand workstation + promotion (SUCRA = 71.0%), treadmill workstation + promotion (SUCRA = 61.6%), seated elliptical + promotion (SUCRA = 56.6%), sit-stand workstation (SUCRA = 50.2%), sit-stand workstation + exercise (SUCRA = 41.7%), promotion (SUCRA = 37.7%), and typical desks (SUCRA = 8.8%).

### Certainty of evidence

The evidence summary for all comparisons is shown in Additional file 3. Of the 10 pairs of direct comparison evidence, one comparison pair was rated as high quality of evidence, four comparison pairs were rated as moderate, four were rated as low, and one was rated as very low. For the evidence of the 18 indirect comparison pairs, two comparison pairs were rated as moderate. Eleven comparison pairs were rated as low, and five comparison pairs were rated as very low.

## Discussion

This network analysis results showed that all interventions produced 28 pairs of comparisons (including 10 pairs of direct comparisons and 18 pairs of indirect comparisons). Based on quantitative statistical effects, multicomponent interventions, treadmill workstation + promotion, sit-stand workstation + promotion, and sit-stand workstation alone were all evidently superior to typical desks. The SUCRA values revealed that multicomponent interventions and sit-stand workstation + promotion had the highest probability of being the optimal intervention. However, the potential effects of the relatively insufficient sample size and number of trials on this conclusion must be acknowledged. The sample size of 5 (21.75%) trials was less than 30, and 7 direct comparisons were based on only one trial. Consequently, the interpretation of the results needs to be based on these circumstances.

Our findings regarding the effects of active workstation interventions are in line with previous systematic reviews and meta-analyses, which consistently demonstrate the benefits of such interventions in reducing sedentary time among office workers [58–61]. However, our study extends the literature by providing a more comprehensive analysis of the effects of single or combination interventions of active workstations and concomitant strategies on occupational sedentary time in office workers. Specifically, we used NMA to quantitatively compare the effects of different active workstation interventions using both direct and indirect evidence, and carefully categorized the interventions based on the type of active workstations and accompanying strategies. This approach allowed us to identify the more effective intervention types and the relative importance of different strategies for reducing sedentary behavior. Furthermore, we used SUCRA values to estimate the probability that each intervention was the best, allowing for a more comprehensive comparison of intervention effectiveness. According to our SUCRA results, multicomponent interventions and sit-stand workstation + promotion had the highest probability of becoming the optimal intervention, followed by treadmill workstation + promotion, seated elliptical + promotion, and sit-stand workstation. This may be because the promotion strategies of multicomponent interventions or active workstation + promotion interventions improve the postural changes of office workers through increasing the usage of active workstations. Moreover, the multicomponent interventions encompass a comprehensive amalgamation of diverse modalities to reduce sedentary behavior including individual strategies such as coaching, promotion, and telephonic support; environmental strategies such as active workstations, prompting posters, and access to a gym; as well as organizational strategies such as education workshops, site visits, and consultations, which highlights the effectiveness of multilevel interventions in the workplace beyond improving posture alone. Therefore, the effect of the multicomponent interventions is better than active workstation + promotion interventions according to the SUCRA value. In contrast, the result for an active workstation intervention alone may decrease after the initial novelty has worn off for the participants [57].

There are several findings worth noting about the quality of the evidence. Eight of the 23 RCTs included in this NMA were rated as high risk of bias due to the low methodological quality, reducing the overall evidence level. In addition, the sample size of some studies was small. The sample size of 12 (52.17%) trials was less than 40; of these, five (21.75%) trials had a sample size of less than 30. These small sample studies influence the overall effect size and level of evidence. For example, we found that the level of evidence for many comparisons was downgraded due to imprecision; the imprecision judgments were based on wide confidence intervals, and the small sizes were the main factor leading to these very wide conference intervals. There methodological limitations underscore the importance of future trials adhering to robust study design principles and implementation guidelines. RCTs with rigorous randomization procedures should be prioritized to minimize bias and increase the validity of findings [62]. Additionally, adopting the Consolidated Standard of Reporting Trials (CONSORT) as the reporting standard can significantly improve study quality and transparency [63]. CONSORT guidelines provide a structured framework for reporting essential aspects of the trial design, conduct, and analysis, enabling readers to evaluate the study’s validity and replicate the findings. To further enhance the scientific quality and reliability of RCTs, investigators should consider utilizing the Cochrane quality assessment tool [19]. This tool allows researcher to conduct a comprehensive self-examination of the study design, hypothesis formulation, data collection and analysis methods, and risk of bias assessment. By critically evaluating these aspects, researchers can identify and address potential limitations, thus strengthen the overall methodological rigor of the trials. Ultimately, the pursuit of more high-quality, large-scale RCTs in the future will be crucial for advancing the field and improving the quality of evidence available.

### Limitations

Our results should be interpreted within the context of the study limitations. First, differences in office workers, such as job type, length of work, the level of postural variation autonomy, and workload, may affect the reliability of the evidence included in this study. Second, we included an RCT by Bergman et. al., that investigated the effectiveness of treadmill workstations compared to typical desks in office workers who were overweight or obese. Our network results showed no statistical differences in performance between sit-stand workstations and treadmill workstations, with treadmill workstations having a lower SUCRA value than sit-stand workstations. Given that obese individuals are generally less physically active than their normal-weight counterparts [64], it is possible that the effect sizes and rankings of the treadmill workstations would change with more evidence. Third, we post-classified and summarized the interventions of the original RCTs, thus potentially introducing some subjective bias. It should be emphasized that different categorizations of interventions might yield different results. However, we felt it was logical and consistent with the ethos of the original objective to assess the effects of different interventions in this NMA. Finally, we did not perform subgroup analyses based on the duration of the interventions and assessment timepoint due to the small number of included studies. However, it is worth noting that the primary assessment in most of the studies included in this NMA were conducted at three months, ranging from one week to six months. This information should be taken into consideration when interpreting our results and drawing conclusions about the effectiveness of active workstations in reducing work-specific sedentary time in office workers.

### Implications for future research

As all studies in this review were from high-income countries, we recommend conducting trials aimed at reducing sitting at work in low- and middle-income countries, where occupational physical inactivity is also increasing [65]. While this NMA found that active workstations alone can effectively reduce work-specific sedentary time in office workers, it is important to consider the potential benefits of incorporating concomitant strategies. Best practice behavior change research suggests that multicomponent interventions, including prompts and visible organizational support, are more successful than workstation alone [66]. Therefore, future research should aim to compare the effectiveness and cost-effectiveness of different combination of active workstation and concomitant strategies to identify the most effective interventions approaches. Furthermore, future research is needed to develop joint interventions that target different characteristics of office workers, such as job type and the level of postural variation autonomy, especially in workplaces with varying levels of physical and cognitive loads across sectors and industries. For instance, as suggested by Hadgraft et. al., (2021), there is low prevalence of many strategies and supports considered both modifiable and low cost and workplaces with different environmental supportive characteristic may require tailored interventions to effectively reduce sedentary behavior. To prevent contamination, we suggest randomizing participants using a cluster randomized design. Locating the intervention and control groups at different sites is beneficial to reducing contamination, since participants in the control group are likely to be less sedentary due to the influence of the intervention group in the same office. Future research would benefit from adding a detailed description of the active workstation intervention’s functionality, e.g., whether it adjusts up and down automatically, to facilitate more detailed analysis. Notably, a systematic review by Nguyen et. al., (2022) found that interventions targeting sedentary behavior in workplaces, such as active workstation, were likely to be cost-effective [67]. However, the review also highlighted gaps in the economic evaluation of interventions and the measurement of sedentary behavior. Importantly, the authors reported that physical environmental changes the installment of active workstations were the key cost driver of interventions. Therefore, future studies should incorporate cost-effectiveness analyses to enable stakeholders and decision-makers to make informed decisions about the appropriateness of a given intervention’s cost in relation to its improvements in health and work-related outcomes, taking into consideration the variations in cost between different workstation models.

## Conclusions

Compared to typical desks, multicomponent interventions, sit-stand workstation + promotion, treadmill workstation + promotion, and sit-stand workstations might be more effective in reducing work-specific sedentary time in office workers. The first two of these interventions are most likely to be the optimal intervention based on SUCRA results. Furthermore, multicomponent interventions and active workstation with promotion yielded better results in reducing work-specific sedentary time compared with active workstation alone. However, the overall certainty of the evidence was low. More high-quality, large-scale, cluster RCTs are needed.

## Supplementary Information

**Additional file 1. **Detailed search strategy.

**Additional file 2. **Node splitting test for inconsistency.

**Additional file 3. **The evidence findings for all comparisons.

## Data Availability

The datasets and any other materials of our study are available from corresponding author on request.

## Abbreviations

RCT: Randomized controlled trial
NMA: Network meta-analysis
SD: Standard deviation
SMD: Standard mean difference
CI: Confidence interval
SUCRA: Surface under the cumulative ranking curve
GRADE: The grading of recommendations, assessment, development, and evaluation approach
CENTRAL: Cochrane Central Register of Controlled Trials
CONSORT: Consolidated Standard of Reporting Trials
