# Supplementary material for: The effects of active workstations on reducing work-specific sedentary time in office workers: a network meta-analysis of 23 randomized controlled trials
Source: Int J Behav Nutr Phys Act. 2023 Jul 27;20:92. doi: 10.1186/s12966-023-01467-5 (PMC10375647; doi:10.1186/s12966-023-01467-5)
Supplement: Supplementary file 1 — Additional file 1. Detailed search strategy. [file 12966_2023_1467_MOESM1_ESM.docx]

**Additional file 1 Detailed search strategy**

**Database: PubMed <17 May 2022>**

Search Startegy:

| **#** | **Searches** | **Results** |
| --- | --- | --- |
| 1 | Occupation [Mesh] | 36,201 |
| 2 | Workplace [Mesh] | 28,146 |
| 3 | “Occupational Groups” [Mesh] | 695,024 |
| 4 | Occupation* [Title/Abstract] | 188,943 |
| 5 | Workplace* [Title/Abstract] | 51706 |
| 6 | Employe* [Title/Abstract] | 474,000 |
| 7 | Office* [Title/Abstract] | 101,255 |
| 8 | (Work-site* [Title/Abstract]) OR (Worksite* [Title/Abstract]) | 4,988 |
| 9 | Worker* [Title/Abstract] | 216,861 |
| 10 | Staff* [Title/Abstract] | 198,280 |
| 11 | White-collar* [Title/Abstract] | 2112 |
| 12 | OR/1-11 | 1,656,709 |
| 13 | “Sedentary Behavior” [Mesh] | 12,927 |
| 14 | “Sitting Position” [Mesh] | 1,355 |
| 15 | “Screen Time” [Mesh] | 1,077 |
| 16 | Sedentary [Title/Abstract] | 37,853 |
| 17 | Sitting [Title/Abstract] | 26,122 |
| 18 | (Inactivity [Title/Abstract]) OR (Inactive [Title/Abstract]) | 118,742 |
| 19 | (“Physical Activity” [Title/Abstract]) OR (“Physically Active” [Title/Abstract]) | 142,534 |
| 20 | (“Screen Time” [Title/Abstract]) OR (“Screen Behavior” [Title/Abstract]) OR (“Screen Behaviour” [Title/Abstract]) OR (“Screen Use” [Title/Abstract]) OR (“Screening Time” [Title/Abstract]) OR (“Screening Behavior” [Title/Abstract]) OR (“Screening Behaviour” [Title/Abstract]) OR (“Screening Use” [Title/Abstract]) | 5,729 |
| 21 | (“Computer Time” [Title/Abstract]) OR (“Internet Time” [Title/Abstract]) OR (“Computer Use” [Title/Abstract]) OR (“Internet Use” [Title/Abstract]) OR (“Computer Usage” [Title/Abstract]) OR (“Internet Usage” [Title/Abstract]) | 6,391 |
| 22 | OR/13-21 | 303,361 |
| 23 | “Randomized controlled trial” [Mesh] | 161,468 |
| 24 | Random* [Title/Abstract] | 1,354,758 |
| 25 | (Blind* [Title/Abstract]) OR (Singleblind* [Title/Abstract]) OR (Doubleblind* [Title/Abstract]) OR (Trebleblind* [Title/Abstract]) OR (Tripleblind* [Title/Abstract]) | 336,700 |
| 26 | (RCT* [Title/Abstract]) OR (Control* [Title/Abstract]) | 4,449,913 |
| 27 | (“Clinical Trial” [Title/Abstract]) OR (“Clinical Trials” [Title/Abstract]) OR (“Clinical Study” [Title/Abstract]) OR (“Clinical Studies” [Title/Abstract]) OR (“Intention to Treat Analysis” [Title/Abstract]) | 616,022 |
| 28 | OR/23-27 | 5,652,319 |
| 29 | 12 AND 22 AND 28 | 7091 |

**Database: Cochrane Library <17 May 2022>**

Search Strategy:

| **#** | **Searches** | **Results** |
| --- | --- | --- |
| 1 | MeSH descriptor: [Occupations] explode all trees | 198 |
| 2 | MeSH descriptor: [Workplace] explode all trees | 980 |
| 3 | MeSH descriptor: [Occupational Groups] explode all trees | 12,409 |
| 4 | (Occupation*):ti,ab,kw | 13,458 |
| 5 | (Workplace*):ti,ab,kw | 3,490 |
| 6 | (Employe*):ti,ab,kw | 19.679 |
| 7 | (Office*):ti,ab,kw | 11,834 |
| 8 | (Work-site*):ti,ab,kw OR (Worksite*):ti,ab,kw | 1,062 |
| 9 | (Worker*):ti,ab,kw | 12,778 |
| 10 | (Staff*):ti,ab,kw | 23,408 |
| 11 | (White-collar*):ti,ab,kw | 107 |
| 12 | OR/1-11 | 81,056 |
| 13 | MeSH descriptor: [Sedentary Behavior] explode all trees | 1,350 |
| 14 | MeSH descriptor: [Sitting Position] explode all trees | 152 |
| 15 | MeSH descriptor: [Screen Time] explode all trees | 33 |
| 16 | (Sedentary):ti,ab,kw | 8,935 |
| 17 | (Sitting):ti,ab,kw | 9,256 |
| 18 | (Inactivity):ti,ab,kw OR (Inactive):ti,ab,kw | 7,697 |
| 19 | (“Physical Activity”):ti,ab,kw OR (“Physically Active”):ti,ab,kw | 39,201 |
| 20 | (“Screen Time”):ti,ab,kw OR (“Screen Behavior”):ti,ab,kw OR (“Screen Behaviour”):ti,ab,kw OR (“Screen Use”):ti,ab,kw OR (“Screening Time”):ti,ab,kw | 659 |
| 21 | (“Screening Behavior”):ti,ab,kw OR (“Screening Behaviour”):ti,ab,kw OR (“Screening Use”):ti,ab,kw | 170 |
| 22 | (“Computer Time”):ti,ab,kw OR (“Computer Use”):ti,ab,kw OR (“Computer Usage”):ti,ab,kw | 233 |
| 23 | (“Internet Time”):ti,ab,kw OR (“Internet Use”):ti,ab,kw OR (“Internet Usage”):ti,ab,kw | 222 |
| 24 | OR/13-23 | 56,805 |
| 25 | MeSH descriptor: [Randomized Controlled Trial] explode all trees | 118 |
| 26 | (Random*):ti,ab,kw | 1,167,879 |
| 27 | (Blind*):ti,ab,kw OR (Singleblind*):ti,ab,kw OR (Doubleblind*):ti,ab,kw OR (Trebleblind*):ti,ab,kw OR (Tripleblind*):ti,ab,kw | 421,341 |
| 28 | (RCT*):ti,ab,kw OR (Control*):ti,ab,kw | 1,142,269 |
| 29 | (“Clinical Trial”):ti,ab,kw OR (“Clinical Trials”):ti,ab,kw OR (“Clinical Study”):ti,ab,kw OR (“Clinical Studies”):ti,ab,kw OR (“Intention to Treat Analysis”):ti,ab,kw | 625,096 |
| 30 | OR/#25-29 | 1,278,119 |
| 31 | #12 AND #24 AND #30 | 4,706 |

**Database: Web of Science <11 May 2022>**

All Web of Science Core Collection

Search Strategy:

| # | Searches | Results |
| --- | --- | --- |
| 1 | TS=(Occupation*) | 239,383 |
| 2 | TS=(Workplace*) | 95,475 |
| 3 | TS=(Employe*) | 1,149,967 |
| 4 | TS=(Office*) | 176,638 |
| 5 | (TS=(Work-site*)) OR TS=(Worksite*) | 5,622 |
| 6 | TS=(Worker* ) | 328,117 |
| 7 | TS=(Staff*) | 213,256 |
| 8 | TS=(White-collar*) | 4,077 |
| 9 | OR/1-8 | 1,971,789 |
| 10 | TS=(Sedentary) | 47,604 |
| 11 | TS=(Sitting) | 81,274 |
| 12 | (TS=(Inactivity)) OR TS=(Inactive) | 127,044 |
| 13 | (TS=(“Physical Activity”)) OR TS=(“Physically Active”) | 225,836 |
| 14 | (((((((TS=(“Screen Time”)) OR TS=(“Screen Behavior”)) OR TS=(“Screen Behaviour”)) OR TS=(“Screen Use”)) OR TS=(“Screening Time”)) OR TS=(“Screening Behavior”)) OR TS=(“Screening Behaviour” )) OR TS=(“Screening Use”) | 6,976 |
| 15 | (((((TS=(“Computer Time”)) OR TS=(“Internet Time”)) OR TS=(“Computer Use”)) OR TS=(“Internet Use”)) OR TS=(“Computer Usage”)) OR TS=(“Internet Usage”) | 17,713 |
| 16 | OR/10-15 | 454,341 |
| 17 | TS=(Random*) | 2,172,167 |
| 18 | ((((TS=(Blind*)) OR TS=(Singleblind*)) OR TS=(Doubleblind*)) OR TS=(Trebleblind*)) OR TS=(Tripleblind*) | 534,642 |
| 19 | (TS=(RCT*)) OR TS=(Control*) | 7,116,232 |
| 20 | ((((TS=(“Clinical Trial”)) OR TS=(“Clinical Trials”)) OR TS=(“Clinical Study”)) OR TS=(“Clinical Studies”)) OR TS=(“Intention to Treat Analysis”) | 620,314 |
| 21 | OR/17-20 | 8,998,056 |
| 22 | #9 AND #16 AND #21 | 8,682 |

**Database: EMBASE <11 May 2022>**

Search Strategy:

| **#** | **Searches** | **Results** |
| --- | --- | --- |
| 1 | 'occupation'/exp OR occupation | 433,383 |
| 2 | 'workplace'/exp OR 'workplace' | 79,633 |
| 3 | 'employee'/exp OR 'employee' | 47,353 |
| 4 | 'office'/exp OR 'office' | 341,296 |
| 5 | 'worker'/exp OR 'worker' | 143,439 |
| 6 | 'staff'/exp OR staff | 380,388 |
| 7 | 'white collar worker'/exp OR 'white collar worker' | 702 |
| 8 | occupation:ti,ab,kw OR occupational:ti,ab,kw | 227,650 |
| 9 | workplace:ti,ab,kw OR workplaces:ti,ab,kw | 64,021 |
| 10 | employee:ti,ab,kw OR employees:ti,ab,kw | 67,951 |
| 11 | office:ti,ab,kw OR offices:ti,ab,kw | 115,383 |
| 12 | 'work site':ti,ab,kw OR 'work sites':ti,ab,kw OR worksite:ti,ab,kw OR worksites:ti,ab,kw | 5,836 |
| 13 | worker:ti,ab,kw OR workers:ti,ab,kw | 264,089 |
| 14 | staff:ti,ab,kw | 263,290 |
| 15 | 'white collar worker':ti,ab,kw | 2,402 |
| 16 | OR/1-15 | 1,550,665 |
| 17 | 'sedentary lifestyle'/exp OR 'sedentary lifestyle' | 22,293 |
| 18 | 'sitting'/exp OR 'sitting' | 47,845 |
| 19 | 'inactivity'/exp OR 'inactivity' | 26,676 |
| 20 | 'physical activity'/exp OR 'physical activity' | 571,073 |
| 21 | 'screen time'/exp OR 'screen time' | 4,963 |
| 22 | sedentary:ti,ab,kw | 49,685 |
| 23 | sitting:ti,ab,kw | 36,953 |
| 24 | inactivity:ti,ab,kw OR inactive:ti,ab,kw | 145,112 |
| 25 | 'physical activity':ti,ab,kw OR 'physically active':ti,ab,kw | 191,966 |
| 26 | 'screen time':ti,ab,kw OR 'screen behavior':ti,ab,kw OR 'screen behaviour':ti,ab,kw OR 'screen use':ti,ab,kw OR 'screening time':ti,ab,kw OR 'screening behavior':ti,ab,kw OR 'screening behaviour':ti,ab,kw OR 'screening use':ti,ab,kw | 7,094 |
| 27 | 'computer time':ti,ab,kw OR 'internet time':ti,ab,kw OR 'computer use':ti,ab,kw OR 'internet use':ti,ab,kw OR 'computer usage':ti,ab,kw OR 'internet usage':ti,ab,kw | 8,045 |
| 28 | OR/17-27 | 772,198 |
| 29 | 'randomized controlled trial'/exp OR 'randomized controlled trial' | 982,996 |
| 30 | 'randomized controlled trial':ti,ab,kw | 134,025 |
| 31 | randomized:ti,ab,kw | 903,262 |
| 32 | blind:ti,ab,kw OR blinds:ti,ab,kw OR blinded:ti,ab,kw OR blinding:ti,ab,kw OR singleblind:ti,ab,kw OR doubleblind:ti,ab,kw OR trebleblind:ti,ab,kw OR tripleblind:ti,ab,kw | 435,403 |
| 33 | rct:ti,ab,kw OR rcts:ti,ab,kw OR controlled:ti,ab,kw | 1,212,526 |
| 34 | 'clinical trial':ti,ab,kw OR 'clinical trials':ti,ab,kw OR 'clinical study':ti,ab,kw OR 'clinical studies':ti,ab,kw OR 'intention to treat analysis':ti,ab,kw | 888,099 |
| 35 | OR/29-34 | 2,752,868 |
| 36 | #16 AND #28 AND #35 | 6,645 |
