# Supplementary material for: The effects of active workstations on reducing work-specific sedentary time in office workers: a network meta-analysis of 23 randomized controlled trials
Source: Int J Behav Nutr Phys Act. 2023 Jul 27;20:92. doi: 10.1186/s12966-023-01467-5 (PMC10375647; doi:10.1186/s12966-023-01467-5)
Supplement: Supplementary file 2 — Additional file 2. Node splitting test for inconsistency. [file 12966_2023_1467_MOESM2_ESM.docx]

**Additional file 2 Node splitting test for inconsistency**

| **Comparison of interventions** | **Direct** | | **Indirect** | | **Difference** | | **P < \|Z\|** |
| --- | --- | --- | --- | --- | --- | --- | --- |
|  | **Coefficient** | **Standard error** | **Coefficient** | **Standard error** | **Coefficient** | **Standard error** |  |
| Typical desk vs Multicomponent Intervention | -1.464 | 0.364 | -2.328 | 1.699 | 0.863 | 1.736 | **0.619** |
| Typical desk vs Sit-Stand Workstation | -1.146 | 0.306 | -0.857 | 0.741 | -0.288 | 0.801 | **0.719** |
| Typical desk vs Sit-Stand Workstation + Promotion | -1.603 | 0.576 | -1.221 | 0.958 | -0.381 | 1.117 | **0.732** |
| Typical desk vs Treadmill Workstation + Promotion | -0.674 | 0.886 | -1.905 | 0.885 | 1.231 | 1.252 | **0.325** |
| Multicomponent Intervention vs Sit-Stand Workstation | 0.648 | 0.924 | 0.327 | 0.498 | 0.321 | 1.049 | **0.760** |
| Promotion vs Seated Elliptical + Promotion | -0.483 | 0.847 | 1.486 | 63.297 | -1.969 | 63.302 | **0.975** |
| Promotion vs Sit-Stand Workstation + Promotion | -0.744 | 0.880 | -2.001 | 28.299 | 1.257 | 28.312 | **0.965** |
| Sit-Stand Workstation vs Sit-Stand Workstation + Exercise | 0.293 | 0.892 | 2.200 | 63.262 | -1.907 | 63.268 | **0.976** |
| Sit-Stand Workstation vs Sit-Stand Workstation + Promotion | -0.146 | 0.912 | -0.525 | 0.645 | 0.378 | 1.117 | **0.735** |
| Sit-Stand Workstation vs Treadmill Workstation + Promotion | -0.742 | 0.838 | 0.490 | 0.930 | -1.232 | 1.252 | **0.325** |
