# Supplementary material for: The effects of active workstations on reducing work-specific sedentary time in office workers: a network meta-analysis of 23 randomized controlled trials
Source: Int J Behav Nutr Phys Act. 2023 Jul 27;20:92. doi: 10.1186/s12966-023-01467-5 (PMC10375647; doi:10.1186/s12966-023-01467-5)
Supplement: Supplementary file 3 — Additional file 3. The evidence findings for all comparisons. [file 12966_2023_1467_MOESM3_ESM.docx]

**Additional file 3. The evidence findings for all comparisons**

| **Certainty assessment** | | | | | | | **№ of patients** | | **Effect** | **Certainty** |
| --- | --- | --- | --- | --- | --- | --- | --- | --- | --- | --- |
| **№ of studies** | **Study design** | **Risk of bias** | **Inconsistency** | **Indirectness** | **Imprecision** | **Publication bias** | **Intervention-1** | **Intervention-2** | **SMD with 95% CI** |  |
| **Sit-stand workstation vs Typical desk** [1, 4, 9, 11, 12, 17, 18, 22, 23] | | | | | | | | | | |
| **9** | RCT | Serious | Not serious | Not serious | Not serious | Undetected | 155 | 145 | -1.10 (-1.64,-0.56) | Moderate |
| **Multicomponent Intervention vs Typical desk** [8, 10, 13, 14, 18, 21] | | | | | | | | | | |
| **6** | RCT | Not serious | Not serious | Not serious | Not serious | Undetected | 332 | 241 | -1.50 (-2.17,-0.82) | High |
| **Sit-stand workstation + Promotion vs Typical desk** [5, 7, 16] | | | | | | | | | | |
| **3** | RCT | Very serious | Serious | Not serious | Not serious | Undetected | 58 | 60 | -1.49 (-2.42,-0.55) | Very low |
| **Sit-stand workstation + Promotion vs Promotion** [19] | | | | | | | | | | |
| **1** | RCT | Serious | Not Serious | Serious | Not serious | Undetected | 19 | 14 | -0.75 (-2.47,0.98) | Low |
| **Sit-stand workstation vs Sit-stand workstation + Exercise** [15] | | | | | | | | | | |
| **1** | RCT | Not serious | Not serious | Serious | Not serious | Undetected | 13 | 13 | -0.29 (-2.04,1.46) | Moderate |
| **Sit-stand workstation + Promotion vs Sit-stand workstation** [6] | | | | | | | | | | |
| **1** | RCT | Not serious | Not serious | Serious | Serious | Undetected | 15 | 16 | -0.39 (-1.38,0.61) | Low |
| **Treadmill workstation + Promotion vs Sit-stand workstation** [2] | | | | | | | | | | |
| **1** | RCT | Serious | Not serious | Not serious | Not serious | Undetected | 39 | 40 | -0.19 (-1.41,1.02) | Moderate |
| **Multicomponent intervention vs Sit-stand workstation** [18] | | | | | | | | | | |
| **1** | RCT | Not serious | Not serious | Serious | Serious | Undetected | 13 | 13 | -0.40 (-1.23,0.43) | Low |
| **Treadmill workstation + Promotion vs Typical desk** [20] | | | | | | | | | | |
| **1** | RCT | Not serious | Not serious | Serious | Serious | Undetected | 15 | 16 | -1.29 (-2.51,-0.07) | Low |
| **Seated elliptical + Promotion vs Promotion** [3] | | | | | | | | | | |
| **1** | RCT | Not serious | Not Serious | Not serious | Serious | Undetected | 27 | 27 | -0.48 (-2.14,1.18) | Moderate |
| **Promotion vs Sit-stand workstation + Exercise** | | | | | | | | | | |
|  | RCT | Serious | Not serious | Serious | Not serious | Undetected |  |  | 0.06 (-2.58,2.71) | Low |
| **Promotion vs Treadmill workstation + Promotion** | | | | | | | | | | |
|  | RCT | Very serious | Serious | Serious | Not serious | Undetected |  |  | 0.55 (-1.74,2.84) | Very low |
| **Promotion vs Typical Desk** | | | | | | | | | | |
|  | RCT | Serious | Not serious | Serious | Not serious | Undetected |  |  | -0.74 (-2.70,1.22) | Low |
| **Promotion vs Multicomponent Intervention** | | | | | | | | | | |
|  | RCT | Very serious | Serious | Serious | Not serious | Undetected |  |  | 0.76 (-1.31,2.82) | Very Low |
| **Multicomponent Intervention vs Treadmill workstation + Promotion** | | | | | | | | | | |
|  | RCT | Not serious | Not serious | Serious | Serious | Undetected |  |  | -0.21 (-1.59,1.18) | Low |
| **Multicomponent Intervention vs Sit-stand workstation + Promotion** | | | | | | | | | | |
|  | RCT | Serious | Not serious | Serious | Not serious | Undetected |  |  | -0.01 (-1.15,1.13) | Low |
| **Multicomponent Intervention vs Sit-stand workstation + Exercise** | | | | | | | | | | |
|  | RCT | Not serious | Not serious | Serious | Serious | Undetected |  |  | -0.69 (-2.63,1.24) | Low |
| **Sit-stand workstation + Exercise vs Typical desk** | | | | | | | | | | |
|  | RCT | Not serious | Not serious | Serious | Not serious | Undetected |  |  | -0.81 (-2.64,1.02) | Moderate |
| **Treadmill workstation + Promotion vs Sit-stand workstation + Promotion** | | | | | | | | | | |
|  | RCT | Very serious | Serious | Serious | Not serious | Undetected |  |  | 0.20 (-1.31,1.71) | Very low |
| **Treadmill workstation + Promotion vs Sit-stand workstation + Exercise** | | | | | | | | | | |
|  | RCT | Not serious | Not serious | Serious | Not serious | Undetected |  |  | -0.48 (-2.61,1.65) | Moderate |
| **Sit-stand workstation + Promotion vs Sit-stand workstation + Exercise** | | | | | | | | | | |
|  | RCT | Not serious | Not serious | Serious | Serious | Undetected |  |  | -0.68 (-2.69,1.33) | Low |
| **Sit-stand workstation vs Promotion** | | | | | | | | | | |
|  | RCT | Not serious | Not serious | Serious | Serious | Undetected |  |  | -0.36 (-2.35,1.63) | Low |
| **Seated elliptical + Promotion vs Typical desk** | | | | | | | | | | |
|  | RCT | Serious | Not serious | Serious | Not serious | Undetected |  |  | -1.22 (-3.79,1.34) | Low |
| **Seated elliptical + Promotion vs Treadmill workstation + Promotion** | | | | | | | | | | |
|  | RCT | Very serious | Serious | Serious | Not serious | Undetected |  |  | 0.07 (-2.76,2.90) | Very low |
| **Seated elliptical + Promotion vs Sit-Stand workstation + Promotion** | | | | | | | | | | |
|  | RCT | Serious | Not serious | Serious | Not serious | Undetected |  |  | 0.26 (-2.13,2.66) | Low |
| **Seated elliptical + Promotion vs Sit-stand workstation + Exercise** | | | | | | | | | | |
|  | RCT | Serious | Not serious | Serious | Not serious | Undetected |  |  | -0.42 (-3.54,2.71) | Low |
| **Seated elliptical + Promotion vs Sit-stand workstation** | | | | | | | | | | |
|  | RCT | Serious | Not serious | Serious | Not serious | Undetected |  |  | -0.12 (-2.72,2.47) | Low |
| **Seated elliptical + Promotion vs Multicomponent intervention** | | | | | | | | | | |
|  | RCT | Very serious | Serious | Serious | Not serious | Undetected |  |  | -0.27 (-2.92,2.38) | Very Low |

Values correspond to the standardized mean difference (SMD) in work-specific time reduction between intervention 1 and intervention 2; for negative values, intervention 1 may be better for reducing work-specific sitting time (e.g., sit-stand workstations had a work-specific sitting time reduction compared with typical desks; SMD = -1.10 (first line)). The results pertaining to rows with 0 number of studies and no cited articles, are derived through the process of indirect comparison in network meta-analysis.

**References**

1. Alkhajah TA, Reeves MM, Eakin EG, Winkler EAH, Owen N, Healy GN. Sit–Stand Workstations. American Journal of Preventive Medicine [Internet]. 2012 [cited 2022 Sep 6];43:298–303. Available from: https://linkinghub.elsevier.com/retrieve/pii/S0749379712003960

2. Bergman F, Wahlström V, Stomby A, Otten J, Lanthén E, Renklint R, et al. Treadmill workstations in office workers who are overweight or obese: a randomised controlled trial. The Lancet Public Health [Internet]. 2018 [cited 2022 Sep 6];3:e523–35. Available from: https://linkinghub.elsevier.com/retrieve/pii/S2468266718301634

3. Carr LJ, Leonhard C, Tucker S, Fethke N, Benzo R, Gerr F. Total Worker Health Intervention Increases Activity of Sedentary Workers. American Journal of Preventive Medicine [Internet]. 2016 [cited 2022 Sep 6];50:9–17. Available from: https://linkinghub.elsevier.com/retrieve/pii/S0749379715003323

4. Chau JY, Daley M, Dunn S, Srinivasan A, Do A, Bauman AE, et al. The effectiveness of sit-stand workstations for changing office workers’ sitting time: results from the Stand@Work randomized controlled trial pilot. Int J Behav Nutr Phys Act [Internet]. 2014 [cited 2022 Sep 6];11:127. Available from: https://ijbnpa.biomedcentral.com/articles/10.1186/s12966-014-0127-7

5. Chau JY, Sukala W, Fedel K, Do A, Engelen L, Kingham M, et al. More standing and just as productive: Effects of a sit-stand desk intervention on call center workers’ sitting, standing, and productivity at work in the Opt to Stand pilot study. Preventive Medicine Reports [Internet]. 2016 [cited 2022 Sep 6];3:68–74. Available from: https://linkinghub.elsevier.com/retrieve/pii/S2211335515001758

6. Donath L, Faude O, Schefer Y, Roth R, Zahner L. Repetitive Daily Point of Choice Prompts and Occupational Sit-Stand Transfers, Concentration and Neuromuscular Performance in Office Workers: An RCT. IJERPH [Internet]. 2015 [cited 2022 Sep 6];12:4340–53. Available from: http://www.mdpi.com/1660-4601/12/4/4340

7. Dutta N, Koepp G, Stovitz S, Levine J, Pereira M. Using Sit-Stand Workstations to Decrease Sedentary Time in Office Workers: A Randomized Crossover Trial. IJERPH [Internet]. 2014 [cited 2022 Sep 6];11:6653–65. Available from: http://www.mdpi.com/1660-4601/11/7/6653

8. Graves L. Evaluation of sit-stand workstations in an office setting: a randomised controlled trial. 2015;14.

9. Edwardson CL, Yates T, Biddle SJH, Davies MJ, Dunstan DW, Esliger DW, et al. Effectiveness of the Stand More AT (SMArT) Work intervention: cluster randomised controlled trial. BMJ [Internet]. 2018 [cited 2022 Sep 6];k3870. Available from: https://www.bmj.com/lookup/doi/10.1136/bmj.k3870

10. Engelen L, Drayton BA, Young S, Daley M, Milton K, Bauman A, et al. Impact and process evaluation of a co-designed ‘Move More, Sit Less’ intervention in a public sector workplace. WOR [Internet]. 2019 [cited 2022 Sep 6];64:587–99. Available from: https://www.medra.org/servlet/aliasResolver?alias=iospress&doi=10.3233/WOR-193020

11. Gao Y, Nevala N, Cronin NJ, Finni T. Effects of environmental intervention on sedentary time, musculoskeletal comfort and work ability in office workers. European Journal of Sport Science [Internet]. 2016 [cited 2022 Sep 6];16:747–54. Available from: http://www.tandfonline.com/doi/full/10.1080/17461391.2015.1106590

12. Healy GN, Eakin EG, LaMontagne AD, Owen N, Winkler EAH, Wiesner G, et al. Reducing sitting time in office workers: Short-term efficacy of a multicomponent intervention. Preventive Medicine [Internet]. 2013 [cited 2022 Sep 6];57:43–8. Available from: https://linkinghub.elsevier.com/retrieve/pii/S0091743513001138

13. Healy GN, Eakin EG, Owen N, Lamontagne AD, Moodie M, Winkler EAH, et al. A Cluster Randomized Controlled Trial to Reduce Office Workers’ Sitting Time: Effect on Activity Outcomes. Medicine & Science in Sports & Exercise [Internet]. 2016 [cited 2022 Sep 6];48:1787–97. Available from: https://journals.lww.com/00005768-201609000-00019

14. Johnston V, Gane EM, Brown W, Vicenzino B, Healy GN, Gilson N, et al. Feasibility and impact of sit-stand workstations with and without exercise in office workers at risk of low back pain: A pilot comparative effectiveness trial. Applied Ergonomics [Internet]. 2019 [cited 2022 Sep 6];76:82–9. Available from: https://linkinghub.elsevier.com/retrieve/pii/S0003687018307312

15. Ma J, Ma D, Li Z, Kim H. Effects of a Workplace Sit–Stand Desk Intervention on Health and Productivity. IJERPH [Internet]. 2021 [cited 2022 Sep 6];18:11604. Available from: https://www.mdpi.com/1660-4601/18/21/11604

16. MacEwen BT, Saunders TJ, MacDonald DJ, Burr JF. Sit-Stand Desks To Reduce Workplace Sitting Time In Office Workers With Abdominal Obesity: A Randomized Controlled Trial. Journal of Physical Activity and Health [Internet]. 2017 [cited 2022 Sep 6];14:710–5. Available from: https://journals.humankinetics.com/view/journals/jpah/14/9/article-p710.xml

17. Mantzari E, Galloway C, Wijndaele K, Brage S, Griffin SJ, Marteau TM. Impact of sit-stand desks at work on energy expenditure, sitting time and cardio-metabolic risk factors: Multiphase feasibility study with randomised controlled component. Preventive Medicine Reports [Internet]. 2019 [cited 2022 Sep 6];13:64–72. Available from: https://linkinghub.elsevier.com/retrieve/pii/S2211335518302729

18. Neuhaus M, Healy GN, Dunstan DW, Owen N, Eakin EG. Workplace Sitting and Height-Adjustable Workstations. American Journal of Preventive Medicine [Internet]. 2014 [cited 2022 Sep 6];46:30–40. Available from: https://linkinghub.elsevier.com/retrieve/pii/S0749379713005369

19. Parry S, Straker L, Gilson ND, Smith AJ. Participatory Workplace Interventions Can Reduce Sedentary Time for Office Workers—A Randomised Controlled Trial. Earnest CP, editor. PLoS ONE [Internet]. 2013 [cited 2022 Sep 6];8:e78957. Available from: https://dx.plos.org/10.1371/journal.pone.0078957

20. Schuna JM, Swift DL, Hendrick CA, Duet MT, Johnson WD, Martin CK, et al. Evaluation of a Workplace Treadmill Desk Intervention: A Randomized Controlled Trial. Journal of Occupational & Environmental Medicine [Internet]. 2014 [cited 2022 Sep 6];56:1266–76. Available from: https://journals.lww.com/00043764-201412000-00007

21. Stephens SK, Winkler EAH, Eakin EG, Clark BK, Owen N, Moodie M, et al. Temporal features of sitting, standing and stepping changes in a cluster-randomised controlled trial of a workplace sitting-reduction intervention. Int J Behav Nutr Phys Act [Internet]. 2019 [cited 2022 Sep 6];16:111. Available from: https://ijbnpa.biomedcentral.com/articles/10.1186/s12966-019-0879-1

22. Tobin R, Leavy J, Jancey J. Uprising: An examination of sit-stand workstations, mental health and work ability in sedentary office workers, in Western Australia. WOR [Internet]. 2016 [cited 2022 Sep 6];55:359–71. Available from: https://www.medra.org/servlet/aliasResolver?alias=iospress&doi=10.3233/WOR-162410

23. Weatherson KA, Wunderlich KB, Faulkner GE. Impact of a low-cost standing desk on reducing workplace sitting (StandUP UBC): A randomised controlled trial. Applied Ergonomics [Internet]. 2020 [cited 2022 Sep 6];82:102951. Available from: https://linkinghub.elsevier.com/retrieve/pii/S0003687019301693
